# Identification and Functional Analysis of Ras-Related Associated with Diabetes Gene (*rrad*) in *Edwardsiella piscicida*-Resistant Individuals of Japanese Flounder (*Paralichthys olivaceus*)

**DOI:** 10.3390/ijms251910532

**Published:** 2024-10-01

**Authors:** Ying Zhu, Xinsheng Yang, Yingming Yang, Xu Yan, Chao Li, Songlin Chen

**Affiliations:** 1 School of Marine Science and Engineering, Qingdao Agricultural University, Qingdao 266109, China; xinsheng0802@163.com (X.Y.); chaoli@qau.edu.cn (C.L.); 2 State Key Laboratory of Mariculture Biobreeding and Sustainable Goods, Yellow Sea Fisheries Research Institute, Chinese Academy of Fishery Sciences, Qingdao 266071, China; yangym@ysfri.ac.cn (Y.Y.); yanxu0210@mails.qust.edu.cn (X.Y.); chensl@ysfri.ac.cn (S.C.); 3 Laboratory for Marine Fisheries Science and Food Production Processes, Qingdao Marine Science and Technology Center, Qingdao 266237, China

**Keywords:** *rrad*, NF-κB, disease-resistant and disease-susceptible individuals, RNAi, methylation, Japanese flounder

## Abstract

Ras-related associated with diabetes (RRAD) is a member of the Ras GTPase superfamily that plays a role in several cellular functions, such as cell proliferation and differentiation. In particular, the superfamily acts as an NF-κB signaling pathway inhibitor and calcium regulator to participate in the immune response pathway. A recent transcriptome study revealed that *rrad* was expressed in the spleen of disease-resistant Japanese flounder (*Paralichthys olivaceus*) individuals compared with disease-susceptible individuals, and the results were also verified by qPCR. Thus, the present study aimed to explore how *rrad* regulates antimicrobial immunity via the NF-κB pathway. First, the coding sequence of *P. olivaceus rrad* was identified. The sequence was 1092 bp in length, encoding 364 amino acids. Based on *phylogenetic* and structural relationship analyses, *P. olivaceus rrad* appeared to be more closely related to teleosts. Next, *rrad* expression differences between disease-resistant and disease-susceptible individuals in immune-related tissues were evaluated, and the results revealed that *rrad* was expressed preferentially in the spleen of disease-resistant individuals. In response to *Edwardsiella piscicida* infection, *rrad* expression in the spleen changed. In vitro, co-culture was carried out to assess the hypo-methylated levels of the *rrad* promoter in the disease-resistant spleen, which was consistent with the high mRNA expression. The siRNA-mediated knockdown of *rrad* performed with the gill cell line of *P. olivaceus* affected many *rrad*-network-related genes, i.e., *dcp1b*, *amagt*, *rus1*, *rapgef1*, *ralbp1*, *plce1*, *rasal1*, *nckipsd*, *prkab2*, *cytbc-1*, *sh3*, and others, as well as some inflammation-related genes, such as *bal2* and *Il-1β*. In addition, flow cytometry analysis showed that *rrad* overexpression was more likely to induce cell apoptosis, with establishing a link between *rrad*‘s function and its potential roles in regulating the NF-κB pathway. Thus,. the current study provided some clarity in terms of understanding the immune response about rrad gene differences between disease-resistant and disease-susceptible *P. olivaceus* individuals. This study provides a molecular basis for fish *rrad* gene functional analysis and may serve as a reference for in-depth of bacterial disease resistance of teleost.

## 1. Introduction

Ras-related associated with diabetes (RRAD), also called the GTP-binding protein Rad, is a member of the Ras GTPase superfamily [1]. It participates in cell proliferation and differentiation, intracellular vesicular transport, and cytoskeleton structure regulation [2,3,4]. Ras signaling activates downstream series effectors such as RAF, MEK, ERK, and other signaling factors [5,6]. Furthermore, the Ras-like small GTPase RRAD plays a crucial role in the proliferation and differentiation of cells. In terms of disease and immunity, RRAD acts as a glycolytic pathway inhibitor and calcium regulator to participate in the immune response pathway. In addition, as a member of the RGK family of Ras-related small GTPases, RRAD exhibits low intrinsic GTPase activity, which is involved in the CaMKII signaling cascade, thereby interacting with calmodulin and calmodulin-dependent protein kinase II [7,8] and with β-tropomyosin [9]. RRAD negatively regulates NF-κB signaling to inhibit the Warburg effect in lung cancer cells, which is frequently activated in cancer [10,11]. In signal transduction, RRAD promotes the EGFR signaling pathway to mediate STAT3 activity, thereby participating in signal transmission function in T cells as well as in cell proliferation, survival, migration, and differentiation [12,13,14].

In fish, the Ras superfamily members, including Rab, Rac, and Raf, participate in specific immune responses after pathogen infection [15,16,17,18]. However, reports on RRAD, a member of the superfamily, are rare in teleosts. It is known that NF-κB is a dual-direction regulator (activation and inhibition) in the immune response process [19,20,21]. In humans, although RRAD acts as a negative regulatory factor to control NF-κB activation, few studies have studied its role in fish innate immunity. Meanwhile, RRAD has been shown to act as a switching molecule in GTPase activity, as it is longer in length than Ras because of an additional sequence at the NH_2_ and COOH termini. In general, it contains five regions essential for binding guanine nucleotides and acting as a GTPase [22]. It is noteworthy that the DNA methylation of CpG islands located in the promoter region of *RRAD* in spleen tissue frequently inactivates its transcription in different cancer cells, such as nasopharyngeal carcinoma, prostate cancer, cervical carcinoma, lung cancer, breast cancer, and malignant mesothelioma, as part of transcriptional regulation [23,24,25,26,27,28]. These findings suggested that the DNA methylation of the CpG island in the 5’UTR of *RRAD* is the major mechanism that induces transcriptional inactivation [29,30,31]. However, the *RRAD* function involving an immune response triggered by bacterial infection has been rarely reported in teleosts.

A variety of marine fish are found in Asian countries, including *Paralichthys olivaceus* [32]. However, bacterial and viral infection by *Edwardsiella piscicida* can affect *P. olivaceus* growth in aquaculture production [33]. *E. piscicida* is a Gram-negative bacterium that causes serious damage to Japanese flounder. In general, fish have built strong innate immune systems to fight pathogens [34]. *E. piscicida* is an enterobacterium that can infect the internal organs of fish, e.g., the spleen, liver, and kidney. Infection with *E. piscicida* usually leads to systemic hemorrhagic septicemia and enlarged liver cell nuclei, which is an important issue in aquaculture.

A previous study revealed that the expression of *rrad*, which was identified in the transcriptome of the Japanese flounder, was significantly high in the spleen of disease-resistant *P. olivaceus* individuals (DR-*Po*) compared with disease-susceptible *P. olivaceus* individuals (DS-*Po*). Based on homology analysis, this gene was *Po-rrad*. In the present study, the transcriptional profile of the spleen, kidney, intestine, and liver of different *P. olivaceus* types was characterized. In addition, the *rrad* expression profile in immune-related tissues after *E. piscicida* challenge was analyzed. The results highlighted *rrad* mRNA expression and the correlation of its methylation level with its effects on the transcription of immune function genes.

## 2. Results

### 2.1. Sequence and Characteristics of rrad in P. olivaceus

**Figure 1:**
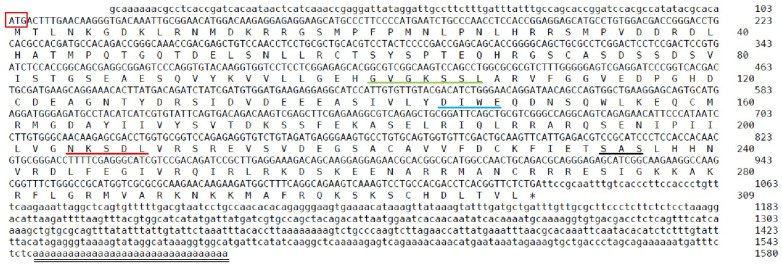
cDNA and predicted amino acid sequences of *Po-rrad*. The small letters show nucleotides, and capital letters denote predicted amino acid sequences. The letters in the red box indicate the start codon (ATG). The double underline marks the poly-A sequence. The black star represents the stop codon (TGA). The conserved domains are shown based on prediction. The green, blue, red, and black line represent G1, G2, G3, and G4 box, respectively.

**Figure 2:**
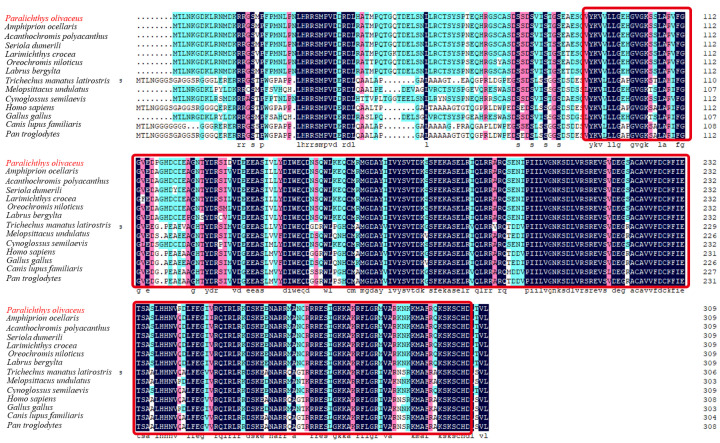
Structural domains and rrad amino acid sequences of *P. olivaceus* and other vertebrates. All sequences were aligned using DNAMAN. The red box represent RGK domain.

**Figure 3:**
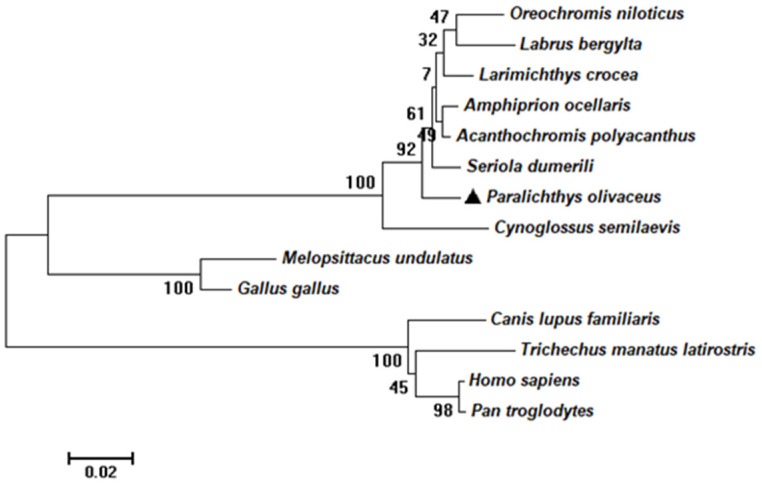
A phylogenetic tree was constructed with the neighbor-joining algorithm in MEGA 4.0. The relative genetic distances are indicated by the scale bar and the branch lengths. The protein sequences of the different species used to build the tree were as follows: *Oreochromis niloticus* rrad (XP_003445756.1), *Labrus bergylta*, rrad (XP_020508528.1), *Larimichthys crocea* rrad (XP_019116206.1), *Amphiprion ocellaris*, rrad (XP_003445756.1), *Acanthochromis polyacanthus*, rrad (XP_022057232.1), *Seriola dumerili* rrad (XP_022623546.1), *Paralichthys olivaceus* rrad (XP_019935401.1), *Cynoglossus semilaevis* rrad (XP_008308414.1), *Melopsittacus undulatus*, RRAD (XP_005152231.1), *Gallus gallus* RRAD (NP_001264535.3), *Canis lupus familiaris* RRAD (XP_038520230.1), *Trichechus manatus latirostris*, RRAD (XP_004371570.1), *Homo sapiens* RRAD (AAB17064.1), and *Pan troglodytes* RRAD (XP_001143391.3). The black triangle represents the target species.

The length of the coding sequence of *rrad* was 1092 bp, encoding a 364-amino-acid protein (Figure 1). According to SMART and DNAMAN prediction, an RGK domain was present in the protein (Figure 2). According to the amino acid sequences, a phylogenetic tree was constructed to assess similarities/differences among fish rrad proteins, including *P. olivaceus* rrad. The rrad protein of Japanese flounder showed a high degree of identity with its orthologs from other teleost species, including fish were clustered in clade I, whereas birds and mammals were grouped in clade II (Figure 3).

### 2.2. Tissue Expression Distribution of rrad

**Figure 4:**
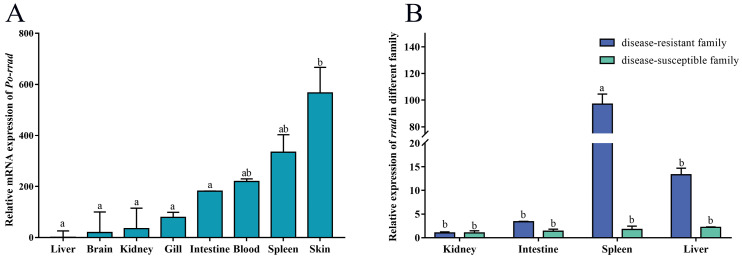
*rrad* expression level in *P. olivaceus* evaluated using qPCR. (**A**) Relative *rrad* mRNA expression in the various tissues of normal fish. (**B**) Relative *rrad* mRNA expression in the immune-related tissues of disease-resistant and disease-susceptible individuals. The mean ± SEM values from three separate individuals (*n* = 3) are shown. The different letters “a” and “b” indicate significant differences (*p* < 0.05).

The tissue expression distribution of *rrad* was assessed in normal fish, and a significant expression was detected in the spleen, blood, and skin, with some expression in other tissues, such as the gills and intestines. In addition, qPCR was used to detect the expression of *rrad* between DR_*Po* and DS_*Po* by assessing the mRNA levels in immune-related tissues (spleen, kidney, intestines, and liver). The results showed a predominant expression in the spleen of DR_*Po*. In other tissues, *rrad* expression was relatively low without any significant difference between DR_*Po* and DS_*Po* (Figure 4).

### 2.3. Expression of rrad in Immune-Related Tissue after E. piscicida Infection

**Figure 5:**
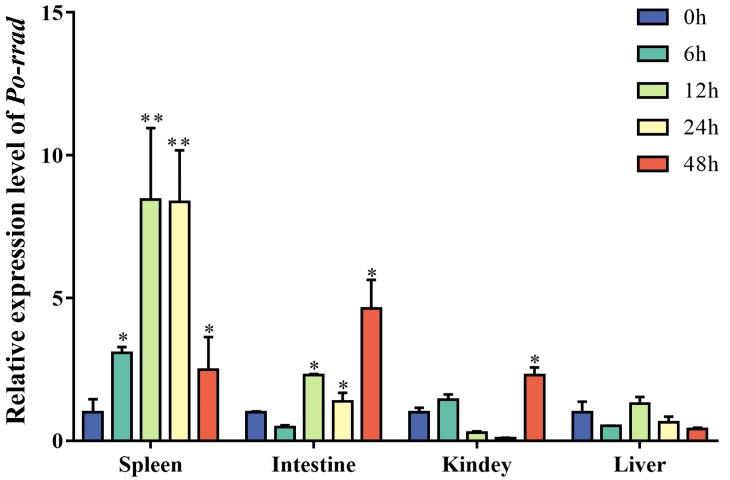
qPCR analysis of *rrad* expression profile in different immune-related tissues (spleen, liver, kidney, and intestines) after *E. piscicida* infection. The results were determined at different time points (0, 6, 12, 24, and 48 h), and PBS was used as the control. The transcription levels were normalized using *β-actin* levels. Data were analyzed using IBM SPSS Statistics 19 with the independent samples t-test. A asterisk stand for a significant difference in comparison with the 0 h group (*p* < 0.05), two asterisks represented significantly different comparison with the 0 h group (*p* < 0.01).

To investigate whether *rrad* participates in the immune response against *E*. *piscicida*, its mRNA expression was detected in the spleen, kidney, liver, intestines, gills, and skin after *E*. *piscicida* infection (from 0 to 48 h). In the spleen, *Po-rrad* was steadily upregulated and then downregulated, with a peak value at 12 h. By contrast, in the liver, *rrad* expression was low, indicating an inconspicuous response to *E*. *piscicida* infection. In the intestines, *rrad* expression increased until it reached a peak at 48 h (Figure 5).

### 2.4. In Vitro Stimulation with PAMPs

**Figure 6:**
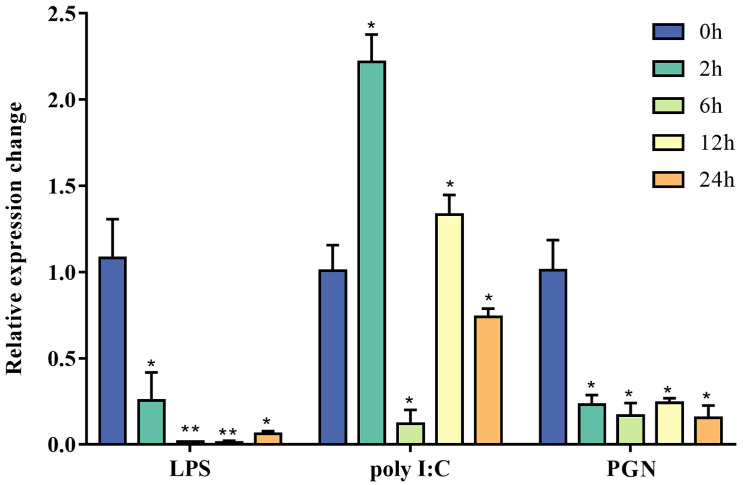
In vitro stimulation of *rrad* in response to LPS, PGN, and poly I:C in the gill cell line of *P. olivaceus*. The data were measured using quantitative RT-PCR and normalized using *β*-actin gene as an internal control. The data are presented as the mean ± standard deviation of three biological replicates. The expression levels with different letters were significantly different, including a asterisk represented significantly different comprared to control (*p* < 0.05), two asterisks represented significantly different comprared to control (*p* < 0.01).

To characterize the immune response pattern of *Po-rrad*, expression changes in *rrad* in gill cells in response to LPS, PGN, and poly I:C were assessed. The results revealed that *Po-rrad* was significantly downregulated from 2 h after exposure to LPS and PGN. In the polyI:C group, it was upregulated at 2 h, rapidly returned to baseline at 12 h, and eventually downregulated at 24 h (Figure 6).

### 2.5. Methylation Analysis of rrad in the Spleen

**Figure 7:**
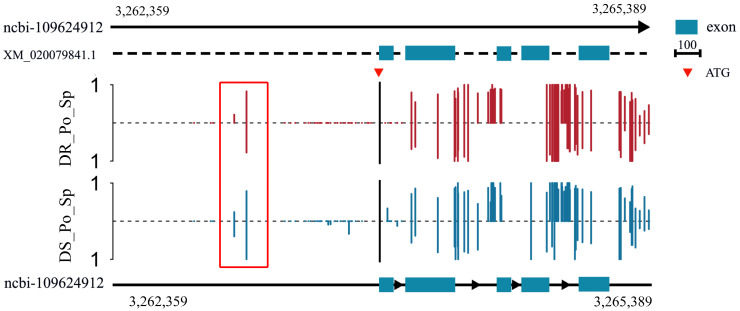
DNA methylation of *rrad* in the promoter and gene body. Methylation differences in the *rrad* promoter and gene body in the spleen tissue of disease-resistant (DR_*Po*_Sp) and disease-susceptible (DS_*Po*_Sp) individuals. Red and blue vertical lines illustrate the methylation level of cytosines, whereas solid rims denote methylation and unmethylation positions, respectively, in disease-resistant individuals, and blue indicates disease-susceptible individuals. The red box is the difference in promoter region methylation between disease-resistant and disease-susceptible individuals.

The methylation analysis of *rrad* in the spleen revealed different methylation levels in the putative promoter and gene body between DR_*Po* and DS_*Po*. The data revealed a relatively slight high degree of methylation in the gene promoter in the spleen of DS_*Po* in contrast with the lowest expression in the spleen of DS_*Po* (Figure 7).

### 2.6. Functional Characterization of rrad Promoter

**Figure 8:**
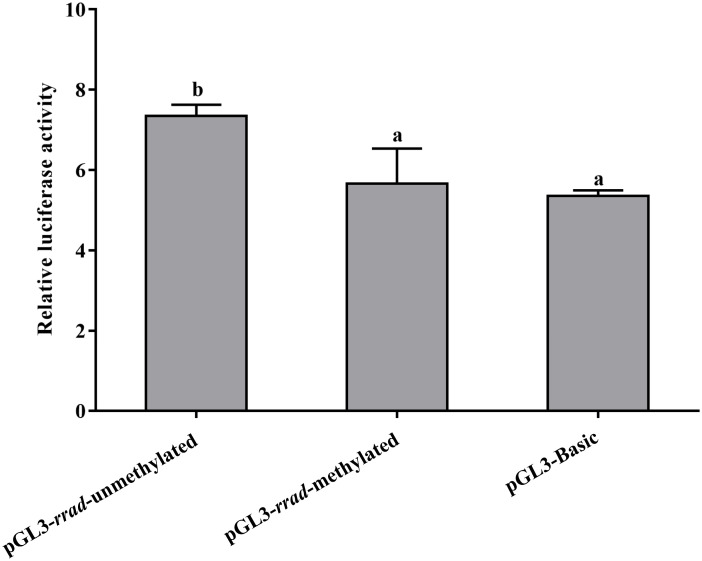
Methylation analysis. Luciferase assays for methylated and unmethylated recombinant plasmids. The *x*-axis shows different recombinant plasmids, and the *y*-axis shows the relative luciferase activity. The letters “a” and “b” indicate significant differences (*p* < 0.05).

To examine the relationship between DNA methylation levels in the promoter region, the 860 bp promoter sequence containing DMRs (differentially methylated regions) was amplified and cloned into the pGL3-basic luciferase reporter vector using in vitro *M.Sss*. In addition, methylated and unmethylated CpG island sequence-containing recombinant plasmids were transfected in HEK 293 T cells. A significantly high firefly/renilla luciferase activity was detected in the pGL3-*rrad*-unmethylated cells compared to the pGL3-*rrad*-methylated and pGL3-basic cells. Thus, the promoter activity was compared with the negative control (pGL3-basic plasmid). In addition, the results indicated that the methylation of the pGL3-rrad plasmids led to a significant repression of promoter activity (*p* < 0.05) (Figure 8).

### 2.7. Luciferase Activity and Flow Cytometry Analyses

**Figure 9:**
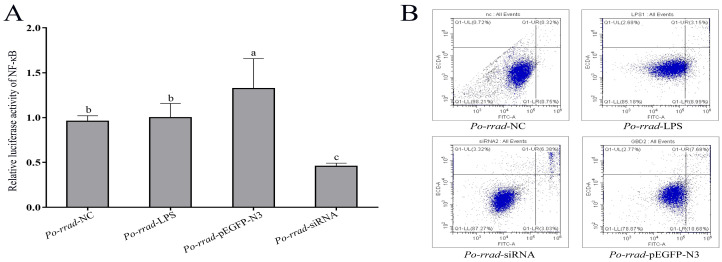
Effect of *rrad* on the transcriptional activity of NF-κB. (**A**) Effect of overexpression and siRNA treatment of *rrad* on the transcriptional activity of NF-κB luciferase reporter gene for 48 h, after which the luciferase activity was measured. (**B**) After co-transfection of *Po-rrad*-pEGFP-N3, *Po-rrad*-siRNA, and LPS with the NF-κB luciferase reporter gene, cells were stimulated for 24 h. The letters “a” “b” and “c” indicate significant differences (*p* < 0.05).

The results of the dual-luciferase reporter assays revealed that *rrad* overexpression and knockdown significantly induced NF-κB activation (Figure 9A). Next, whether the overexpression and RNAi of *rrad* play a role in cell apoptosis was assessed using the fluorescence intensity analysis of Annexin V-FITC/PI. Apoptosis induced by different treatment groups (*rrad*-pEGFP-N3, *rrad*-RNAi, and LPS) is shown in Figure 9B; it was constrained by early apoptosis. The *rrad*-pEGFP-N3 group showed a 10.68% increase in early apoptosis; the rate of late apoptosis increased to 7.68% in the *rrad*-pEGFP-N3 group, 8.99% in the LPS group, and 3.03% in the *rrad*-siRNA group; and the rate of upper apoptosis increased to 3.15% in the *rrad*-pEGFP-N3 group, 3.03% in the LPS group, and 6.38% in the *rrad*-siRNA group. Thus, the results showed that *rrad* overexpression may induce apoptosis.

### 2.8. Protein–Protein Interaction (PPI) Network of rrad

**Figure 10:**
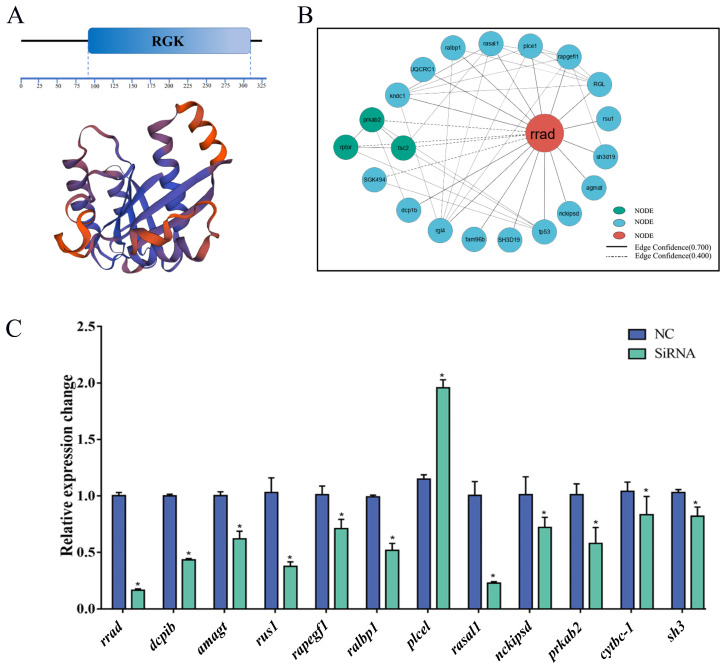
Analysis of PPI interaction and siRNA effects after *rrad* RNAi in *P. olivaceus* gill cells. (**A**) The expression of *rrad*, *dcp1b*, *amagt*, *rsu1*, *rapgef1*, *ralbp1*, *plce1*, *rasal1*, *nckipsd*, *prkab2*, *cytbc-1*, *sh3*, *bcl2*, and *Il-1β* was analyzed in cultured gill cells after RNAi. (**B**) Three-dimensional protein prediction and PPI analysis of rrad. The correlation of proteins was predicted using the STRING 11.0 online database. (**C**) Compared with the control, the mRNA levels of the interaction predictor of *rrad* and other immune response genes, i.e., *dcp1b*, *amagt*, *rsu1*, *rapgef1*, *ralbp1*, *plce1*, *rasal1*, *nckipsd*, *prkab2*, *cytbc-1*, *sh3*, *bcl2*, and *Il-1β*, were detected after RNAi. The stars represented a significant differentce copared to the control group (*p* < 0.05).

According to the PPI network, rrad and its interacting proteins are shown in Figure 10. An analysis of the PPI data revealed a significant enhancement of several signaling pathway factors, such as rasal1, plce1, rgl, rsu1, agmat, nckipsd, tp53, fam96b, and rgl4.

### 2.9. Effect of the Knockdown of rrad and Other Related Genes via siRNA Transfection

An RNAi experiment was performed on *P. olivaceus* gill cells to investigate the possible effects of *rrad* knockdown. Three siRNA locations were designed in exons 5, 6, and 7. A 48 h time point after siRNA transfection was chosen to measure *rrad* expression. Because the results revealed that siRNA-3 had a higher transcription efficiency than siRNA-nc, qPCR was performed on the cells transfected with siRNAs along with siRNA-nc as the control. The associated genes, including the predicted *rrad* interaction network regulation-related genes of *dcp1b*, *amagt*, *rus1*, *rapgef1*, *ralbp1*, *plce1*, *rasal1*, *nckipsd*, *prkab2*, *cytbc-1*, and *sh3*, were assessed (Figure 10C). The results showed that *bcl2* and *Il-1β*, which are associated with inflammation, were upregulated compared with the control after the *rrad* RNAi treatment.

## 3. Discussion

RRAD belongs to a class of Ras-related GTPases that play a crucial role in cell proliferation and differentiation. Recent studies have suggested that RRAD may suppress some types of human cancer (breast, lung, and ovary) [26,35], probably by inhibiting NF-κB. This mechanism, which was confirmed through its interaction with p65 [36,37], was shown to inhibit the Warburg effect and promote immunological functions in cancer cells in mammals [38]. NF-κB signaling requires optimal activation; however, mechanisms through which bacteria interfere with host NF-κB signals remain unknown [39]. In teleosts, the immune-related function of *rrad* is poorly understood, and few studies have assessed the potential function of *rrad* regulation in the NF-κB pathway and downstream factors in teleosts.

In the present study, the full-length *Po-rrad* sequence from *P. olivaceus*, which encodes a 309-amino-acid protein containing the GTP/Mg^2+^ binding site including G1, G2, G3, and G4 box domains, was cloned. The Rad protein has five highly conserved GTPase domains G1–G4, which are characteristic of Ras-related proteins. Meanwhile, rrad has several nonconservative amino acids in the G1, G2, and G3 domains that may affect its GTPase function [40]. Furthermore, there are several characteristics that distinguish rrad from other Ras-related GTPases, including the lack of characteristic domains such as prenylation motifs, a GTP-binding domain, and NH2- and COOH-terminal extensions [41]. Although there are no NH2- and COOH-terminal extensions, a GTP-binding domain exists in the *rrad* of *P. olivaceus*. In addition, multiple *rrad* forms exist in different species. In this study, the phylogenetic tree revealed that the rrad protein in *P. olivaceus* was highly similar to that of other bony fishes (Figure 2 and Figure 3), whereas mammals clustered into another branch. This result suggested that *P. olivaceus rrad* is closely related to other teleosts at the evolutionary level.

In the current study, *Po-rrad* was expressed ubiquitously in almost all the examined tissues. In particular, *Po-rrad* expression was the highest in the skin, blood, and spleen. In addition, the qPCR results showed that *Po-rrad* was preferentially expressed in the spleen tissue of DR-*Po* and that its expression was not significantly different in other immune-related tissues, e.g., the liver, gill, intestines, and kidney. It is noteworthy that, as a kind of epigenetic modification, the promoter of *rrad* was hypermethylated, which may have led to a relatively low expression in DS-*Po*. In general, DNA methylation can repress transcriptional activity [42]. Therefore, hypermethylation is likely to be an important factor affecting the low expression of *rrad* in disease-susceptible individuals. Meanwhile, *rrad* was significantly induced in the spleen after *E. piscicida* challenge, with a slight expression change in other immune-related tissues (intestines, kidney, and liver). In humans, *rrad* is most highly expressed in the skeletal muscle, heart, and lung [1], which may inhibit smooth muscle vascular migration and attachment. Meanwhile, *rrad* expression in the spleen and intestine tissues of fish was affected after *E. piscicida* infection. A similar result was obtained in a previous study that revealed that *rrad* expression could respond to oxidative stress induced following *Streptococcus pneumoniae* challenge in mice [43]. In fact, together with its regulatory and effector molecules, *rrad* acts as a tumor suppressor gene in the immune system, with its intermediaries involved in the complex signaling pathways that control cellular processes and survival [44]. Above all, the results indicated that *rrad* may be a key factor in immune responses against infection in teleosts.

To investigate the effect of *rrad*, transcriptional activity was detected using a luciferase assay, which showed a correlation with NF-κB regulation in *P. olivaceus*. Rrad has been identified as a p53-regulated gene that influences NF-κB transcription. Other studies have revealed the effect of IκBα on NF-κB regulation. In *Oncorhynchus mykiss* and *Ctenopharyngodon idellus*, the binding between IκBα and p65 induced NF-κB regulation in cellular homeostasis [20,45,46]. In addition, the PIAS and COMMD of *Branchiostoma belcheri* were identified as the negative regulators of NF-κB signaling involved in the inflammatory response process [47,48]. Thus, *rrad* plays a potential crucial role in regulating the positive movement of the downstream critical factor NF-κB. Meanwhile, by regulating gene expression in response to infection or inflammation, although NF-κB participates in their proliferation, its role is still unclear [49]. It is known that NF-κB is a transcription factor that promotes cell proliferation, differentiation, and survival [50]. To study the effect of *rrad* on cell apoptosis, flow cytometry analysis was used to detect the NF-κB-induced apoptosis rate after *rrad* knockdown or overexpression for 48 h in a gill cell line. Among its multitude of cellular functions, *rrad* is involved in apoptosis, tumor cell division, motility, and energy metabolism along with its interaction with multiple downstream effectors [44]. This study’s results indicated that *rrad* may be a key regulator of NF-κB that participates in immune response processes.

The in vitro knockdown of *rrad* led to the upregulation of the inflammation-related factors *bal2* and *Il-1β*. Interleukin-1β, a proinflammatory cytokine, is essential for controlling the innate immune response, inflammatory process, and immune homeostasis [51], and it is upregulated in host antibacterial signaling [52]. Moreover, apoptosis is regulated during infection via the B-cell lymphoma (Bcl2) proteins, and cellular stress can trigger apoptosis [53]. Studies have also shown that *rrad* inhibits the mTOR pathway mediating the formation of the Bcl-2 complex to prevent autophagy [54]. After the in vitro knockdown of *rrad*, certain PPI-network-related genes regulated by *rrad*, such as *rapgef1* and *rasal1*, were strongly downregulated. An early mucosal response in blue catfish (*Ictalurus furcatus*) is facilitated by rapgef1, a guanine nucleotide exchange factor that interacts with c-Abl in the cytoskeleton [55]. Meanwhile, RAS protein activator like-1 (rasal1) is an RAS/mitogen-activated protein kinase produced in response to growth factor stimulation that serves as a tumor suppressor in cancer cells [56]. Thus, it can be hypothesized that *rrad* participates in the immune response against inflammation caused by bacterial pathogens.

The DNA methylome pattern of *rrad* showed hypomethylated modification sites in the CpG island in DR-*Po* individuals. In addition, methylation was negatively correlated with gene transcription in the spleen tissue. The luciferase reporter assay revealed that high methylation in *Po*-*rrad* promoter could significantly suppress transcriptional activity (*p* < 0.05). It is noteworthy that *rrad* promoter hypermethylation occurs with a concomitant *rrad* expression loss in human cancer tissue (e.g., ovarian, lung, and breast) [41]. Thus, it is evident from these studies that *rrad* DNA methylation is inversely associated with the expression of tumor suppressor factors in the immune response. Research has shown that *rrad* in the 5’UTR region of the CpG island leads to methylation modification, which is the major mechanism that induces the transcriptional inactivation of tumor suppressor genes [57]. These results suggest that immune-related *Po-rrad* expression results from epigenetic regulation in disease-susceptible individuals.

In summary, *rrad* was identified from *P. olivaceus*, and *Po-rrad* expression was the highest in the skin, blood, and spleen. It was preferentially expressed in the spleen tissue of DR-*Po* individuals. Meanwhile, *E. piscicida* bacterial infection led to increased *rrad* expression in the spleen. Moreover, the methylation level in the *rrad* promoter was negatively associated gene expression, while, the luciferase assay showed that *rrad* may have a potential regulatory effect on NF-κB, but the specific regulatory mode and action subunit need to be further studied. The identification of *rrad* provided important data for understanding its important role in the immune response *E. piscicida* bacterial infection in *P. olivaceus*. Future research should focus on elucidating the precise molecular mechanisms by which *rrad* influences the NF-κB pathway, exploring its potential interactions with other immune-related genes, and investigating its broader implications in host–pathogen interactions and disease resistance in flatfish.

## 4. Materials and Methods

### 4.1. Fish Sample Preparation

Disease-resistant and disease-susceptible individuals of Japanese flounders identified by the research group of Yellow Sea Aquatic Product Co., Ltd., Yantai, China, were used in this study. According to Chen et al., family establishment is the most common method for establishing families, and this is described in [58]. In this study, established families were selected for the challenge experiment using *E. piscicida*. For disease resistance, the 17L1178 family, with a final survival rate of 87.76%, was considered the disease-resistant family (DR-*Po*). By contrast, the 17L1726 family, with a final survival rate of 10.15%, was considered the disease-susceptible family (DS-*Po*). Six individuals from each family were selected for RNA extraction and sequencing. In addition, a preliminary challenge experiment was performed to determine the concentration of bacteria required for a formal challenge assay as well as to isolate *E. piscicida* from symptomatic fish for biochemical confirmation. Fish used in the experiment were divided into a control group and six experimental groups in 30 L large tanks. Fish in the experimental group were cultured in 1 × 10^7^ CFU/mL *E. piscicida* for 2 h. After bacterial challenge, nine 1-year-old fish were randomly selected at each infection time point (2, 6, 12, 24, and 48 h) for RNA extraction and immune-related tissue (liver, brain, kidney, gills, intestines, blood, spleen, and skin) collection. The tissues were directly frozen with liquid nitrogen and then stored at −80 °C until use.

### 4.2. Gene Cloning and cDNA Synthesis

Total RNA was extracted using the TRIzol reagent (Invitrogen, Carlsbad, CA, USA) according to the manufacturer’s protocol. A Reverse Transcriptase M-MLV Kit was used for cDNA synthesis with 1 μg of total RNA (TaKaRa, Dalian, China) as per the manufacturer’s instructions. RACE-ready cDNA was synthesized from compounded spleen and kidney tissues using an SMRT^TM^ RACE cDNA amplification kit (Invitrogen, Carlsbad, CA, USA) and following the manufacturer’s instructions. For 5′- and 3′-RACE PCR amplification, nested primers (rrad-NGSP-S: GACACTTCTTTTCTTTGGCCTCTGAA, rrad-NGSP-A:5′-TACACCGGAACATCCGT-3′) were used to first perform touchdown PCR as follows: 94 °C for 3 min, followed by 25 cycles of 94 °C for 50 s, then five cycles of 68 °C for 28 s, a 2 °C temperature reduction every five cycles, and 72 °C for 5 min. Next, final amplification was performed as follows: 35 cycles of 94 °C for 40 s, 58 °C for 55 s, and 72 °C for 5 min, followed by elongation at 72 °C for 10 min. Finally, 1.0% agarose gel was used to separate the amplicons, and a DNA purification kit was used for their purification (Tiangen, Beijing, China). The product was subcloned into a pEASY-T1 vector (TaKaRa, Dalina, China) and sequenced.

### 4.3. Bioinformatic Analysis

The 5′- and 3′-RACE fragments were assembled using the DNASTAR version 6.1.0 (DNASTAR, Madison, WI, USA), and the amino acid sequence was aligned via DNAMAN version 5.2.2. Based on the neighbor-joining method, MEGA version 7.0 was used to construct phylogenetic trees. To obtain the phylogenetic relationship of *rrad*, a tree was constructed using the maximum likelihood method via the software IQ-tree 2.

### 4.4. Quantitative Real-Time PCR (qPCR) Analysis

As described previously, qPCR was used to determine the *rrad* expression level in various immune-related tissues (liver, kidney, gills, intestines, blood, spleen, and skin) [59]. Gene expression quantification was performed on an ABI 7500 Fast Detection system with SYBR green Master Mix (TaKaRa, Dalian, China), and *β-actin* was used as the control (Applied Biosystems, Waltham, MA, USA). Three samples were prepared, and triplicate tests were performed per sample. The qPCR procedure was as follows: 40 cycles of 94 °C for 5 s and 60 °C for 35 s. The transcription levels were analyzed using the comparative 2^−△△Ct^ method [60], and the data are presented as mean ± standard deviation from three samples using three parallel replicates. The data were analyzed using SPSS version 19.0, variance was analyzed at the significance level according to Duncan’s post hoc test, and the significance level was set at *p* < 0.05.

### 4.5. Effect of rrad on the Survival Integrity of Target Bacteria

To detect the *rrad* gene expression patterns in different immune-related tissues after bacterial infection, ten fish were used for each of the experimental challenges for bacterial and viral infections (approximately 10–12 cm in body length). As part of the bacterial challenge examination, flounders were randomly classified into three groups and injected intraperitoneally with 100 μL 1.0 × 10^4^ CFUs of *E. piscicida* and PBS was injected as a control. Different tissues from the fish were collected at certain time points (0, 12, 24, and 48 h post injection). The experiments were performed in triplicate. The qPCR procedure was using a 7500 Fast real-time PCR system (Applied Biosystems, USA). Procedures for RNA extraction, RNA quality checking, cDNA transcription, and qPCR reaction and melting curve analysis were conducted following the method of Zhu et al. [61]. *β-actin* was used as the internal control. The 2^−ΔΔCt^ method was used to calculate the relative gene expression fold changes. Simultaneously, data were analyzed using one-way ANOVA followed by Duncan’s multiple comparison test with SPSS version 20.0 (IBM, New York, NY, USA) with a statistical significance of *p* < 0.05.

### 4.6. DNA Methylation Status of rrad in Different Individuals

Using genomic sequences from disease-resistant and disease-susceptible individuals, the *rrad* methylation profile was assessed to evaluate the association of methylation with transcription. A methylation analysis was performed with the 2000 bp upstream sequence of the gene, the gene body, and 500 bp downstream sequence of the gene of two different disease-resistant Japanese flounder individuals. The spleens of each type of 1-year-old fish were collected to extract genomic DNA using a TIANamp Marine Animals DNA kit (Tiangen, Beijing, China) according to the manufacturer’s instructions. Then, the extracted DNA of the two individual types was mixed, and the quality and concentration were measured using a NanoVue™Plus Spectrophotometer (GE Healthcare, Piscataway, NJ, USA). The DNA mixture was modified via an EZ DNA Methylation-Gold Kit™ (Zymo Research, Irvine, CA). After cloning into a PMD18-T vector (Takara, Dalian, China) and culturing in *E. coli* Top10 cells (Tiangen, Beijing, China), eight positive clones were sequenced using BGI. The site-specific methylation measurements at the selected CpG and core promoter sites were performed using the BiQ-Analyzer version 3.0. Then, ANOVA was used to identify differentially methylated regions (DMRs) among the different groups of samples. The BS-seq analysis was conducted based on BS-seq library construction and used the reference genome of *P. olivaceus*. To distinguish methylated C nucleotides (mCs), *p*-values < 0.01 were considered positives. The methylation level of *Po-rrad* (including the *Po-rrad* genomic sequence and 2 kb flanking upstream sequence) between the disease-resistant family (DR-*Po*) and the disease-susceptible family (DS-*Po*) were compared according to our methylome data.

### 4.7. rrad Promoter Activity Analysis

The *SacI* and *XhoI* restriction enzymes were used to construct a recombinant plasmid pEGFP-*rrad*. The CDS (coding sequence) of *rrad* was amplified and subcloned into pEGFP plasmid to transfer to cells for *rrad* gene overexpression in vitro. The pEGFP-*rrad*, *rrad*-siRNA, and LPS were co-transfected with NF-κB luciferase reporter plasmids into HEK 293 T cells to detect the effect of *rrad* gene expression on NF-κB, and NF-κB luciferase reporter plasmids were used as controls with 1.2 g/well Lipofectamine 5000 in a 12-well plate. As an internal reference, 80 ng of pRL-TK plasmid was added per well. An assay kit for dual-luciferase reporter gene detection was used to measure firefly and renilla luciferase activities 48 h after transfection using a Varioskan Flash spectral scanning multimode reader (Thermo, Vantaa, Finland). The experiments were performed in triplicate.

### 4.8. SiRNA Design and rrad Knockdown in Gill Cell Line of P. olivaceus

Three pairs of *rrad*-specific siRNAs were synthesized by Guangzhou Ribobio Co., Ltd. (Ribobio, Guangzhou, China); a nonspecific siRNA served as the negative control. Three replicates of the si-*rrad* and NC groups were performed. In the *P. olivaceus* gill cell line, siRNA was transfected with Lipofectamine 5000 following the manufacturer’s instructions. The effects of interference between si-*rrad* and NC were compared after 48 h using qPCR. The expression levels of *dcp1b*, *amagt*, *rus1*, *rapgef1*, *ralbp1*, *plce1*, *rasal1*, *nckipsd*, *prkab2*, *cytbc-1*, *sh3*, *bcl2*, and *Il-1β* were assessed. The experiments were performed in triplicate.

## Data Availability

All data generated or analyzed during this study is contained within the article.
